# Hyperglycemia and insulin use in patients with COVID-19 and severe hypoxemia allocated to 12 mg vs. 6 mg of dexamethasone: a secondary analysis of the COVID STEROID 2 randomized trial

**DOI:** 10.1186/s13613-025-01512-5

**Published:** 2025-07-15

**Authors:** Clara Lundetoft Clausen, Thomas Bryrup, Christian Leo Hansen, Daniel Faurholt-Jepsen, Alessandra Meddis, Thomas Peter Almdal, Ole Snorgaard, Henrik Løvendahl Jørgensen, Marie Helleberg, Margit Smitt, Christian Aage Warmberg, Klaus Tjelle, Charlotte Suppli Ulrik, Anne Sofie Andreasen, Morten Bestle, Lone Poulsen, Klaus Vennick Marcussen, Lothar Wiese, Marie Warrer Munch, Anders Perner, Rikke Krogh-Madsen, Thomas Benfield

**Affiliations:** 1 Center of Clinical Research and Disruption of Infectious Diseases (CREDID), Copenhagen University Hospital - Amager and Hvidovre, Kettegaard Alle 30, 2650 Hvidovre, Denmark; 2 Department of Infectious Diseases, Copenhagen University Hospital - Amager and Hvidovre, Hvidovre, Denmark; 3 Department of Infectious Diseases, Copenhagen University Hospital – Rigshospitalet, Copenhagen, Denmark; 4 Department of Clinical Medicine, University of Copenhagen, Copenhagen, Denmark; 5 Department of Public Health, Section of Biostatistics, University of Copenhagen, Copenhagen, Denmark; 6 Department of Nephrology and Endocrinology, Copenhagen University Hospital, Rigshospitalet, Copenhagen, Denmark; 7 Department of Immunology and Microbiology, Faculty of Health and Medical Sciences, University of Copenhagen, Copenhagen,, Denmark; 8 Department of Endocrinology, Copenhagen University Hospital – Amager and Hvidovre, Hvidovre, Denmark; 9 Department of Clinical Biochemistry, Copenhagen University Hospital – Amager and Hvidovre, Hvidovre, Denmark; 10 Department of Intensive Care, Copenhagen University Hospital – Rigshospitalet, Copenhagen, Denmark; 11 Department of Neuroanaesthesiology, Copenhagen University Hospital – Rigshospitalet, Copenhagen, Denmark; 12 Department of Anaesthesiology and Intensive Care, Copenhagen University Hospital – Bispebjerg and Frederiksberg, Copenhagen, Denmark; 13 Department of Anaesthesia and Intensive Care, Copenhagen University Hospital – Amager and Hvidovre, Hvidovre, Copenhagen, Denmark; 14 Department of Respiratory Medicine, Copenhagen University Hospital – Amager and Hvidovre, Hvidovre, Copenhagen, Denmark; 15 Department of Anaesthesia and Intensive Care, Copenhagen University Hospital – Herlev and Gentofte, Herlev, Copenhagen, Denmark; 16 Department of Anaesthesia and Intensive Care, Copenhagen University Hospital – North Zealand, Hillerod, Denmark; 17 Department of Anaesthesia and Intensive Care, Zealand University Hospital, Køge, Denmark; 18 Department of Anaesthesia, Zealand University Hospital, Slagelse, Denmark; 19 Department of Infectious Diseases, Zealand University Hospital, Roskilde, Denmark; 20 Centre for Physical Activity Research, Copenhagen University Hospital – Rigshospitalet, Copenhagen, Denmark

**Keywords:** COVID-19, Dexamethasone treatment, Higher and standard dose, Hyperglycemia, Hypoglycemia, COVID-steroid 2 trial, Secondary analysis, Adverse events, Insulin

## Abstract

**Background:**

While dexamethasone has been shown to improve survival in COVID-19, its dose–response relationship with plasma glucose (PG) levels and insulin requirements is poorly understood. This study investigated the impact of 12 mg (higher dose) versus 6 mg (standard dose) of dexamethasone on hyper- or hypoglycemic events and the use of insulin.

**Methods:**

A secondary analysis of a subpopulation of the COVID STEROID 2 trial. Glycemic outcomes were assessed by time-to-event analysis of a hyperglycemic (two PG measurements ≥ 11.1 mmol/L), severe hyperglycemic (PG > 20 mmol/L), hypoglycemic (< 3.8 mmol/L) event or use of insulin, adjusted for age, diabetes status, hospital site, and mechanical ventilation. PG levels were compared before and after treatment allocation with linear mixed models to estimate changes in average PG levels over time.

**Results:**

Of 321 participants, 170 were allocated to the higher dose and 151 to the standard dose of dexamethasone. Time to a hyperglycemic event did not differ between groups, whereas severe hyperglycemic events were more frequent in the higher dose group (36%) than in the standard dose group (31%) with an adjusted subdistributional hazard ratio of 1.76 (95% CI [1.22–2.54], p = 0.003). Insulin use and hypoglycemic events did not differ between groups. The higher vs. standard dose group had an average PG increase of 0.5 mmol/L (95% CI [− 0.2 to 1.4], p = 0.149).

**Conclusion:**

Higher vs. standard doses of dexamethasone were associated with a higher incidence of severe hyperglycemia in patients with COVID-19 and severe hypoxemia, but the average increase in PG was similar between groups.

**Supplementary Information:**

The online version contains supplementary material available at 10.1186/s13613-025-01512-5.

## Background

Despite the widespread use of corticosteroids in patients with coronavirus disease 2019 (COVID-19) requiring supplemental oxygen, the dose–response relationship between corticosteroid treatment, plasma glucose (PG) levels, and subsequent insulin requirements remains less studied. Early in the pandemic, it was established that corticosteroids (e.g., dexamethasone 6 mg daily) improved survival in patients with COVID-19 requiring supplemental oxygen [1, 2]. Subsequently, the COVID STEROID 2 trial assessed the effects of 12 mg versus 6 mg of dexamethasone daily for up to 10 days on the number of days alive without life support at 28 days in patients with COVID-19 and severe hypoxemia [3]. While the primary analysis found no statistically significant benefits with the higher dose, the secondary Bayesian analysis indicated a 94% probability of benefit on days alive without life-support and a 95% probability of benefit on mortality with the higher dose [4].

Managing hyperglycemia in patients treated with dexamethasone is challenging and may contribute to a protracted disease course [5]. Infusion of high glucose concentrations leads to increased production of pro-inflammatory cytokines, such as interleukin-6, tumor-necrosis factor, and interferons in both patients with and without diabetes [6–10]. Further, hyperglycemia has been associated with mortality in hospitalized patients with COVID-19 [11–14], probably acting as a surrogate marker of disease severity. Studies on glucose metabolism during different dexamethasone doses have been in either healthy individuals or surgical patients, and while we know that there is a dose–response relationship in healthy individuals, this relationship remains less understood in critically ill patients [15, 16]. The COVID-STEROID 2 trial offers an opportunity to assess this in a real-life setting. We hypothesized that a higher dose of dexamethasone would increase PG levels and the risks of hyperglycemic (PG ≥ 11.1 mmol/L) and severe hyperglycemic (PG ≥ 20 mmol/L) events, as well as more insulin use compared to the standard dose of dexamethasone regardless of baseline diabetes status. Further, we hypothesized that there would be a lower risk of hypoglycemic events in the higher dose group. Thus, this study aimed to assess the differences in the occurrence of hyperglycemic, severe hyperglycemic, or hypoglycemic events, and in time to insulin initiation, following randomization to higher vs. standard doses of dexamethasone. Additionally, we examined changes in PG levels from before to after randomization, as well as glycemic variability in the two groups.

## Methods

### Study design and participants

#### The COVID STEROID 2 trial

The study is a secondary analysis of the COVID STEROID 2 trial, an investerigator-initiated, international, multicentre, parallel-group, blinded, randomized clinical trial [3]. In brief, the trial randomized 1000 adults hospitalized with COVID-19 and severe hypoxemia (requiring at least 10 L of oxygen per minute or mechanical ventilation) from 31 sites in Denmark, India, Sweden, and Switzerland between August 27 of 2020, and May 20 of 2021 in a 1:1 ratio to receive either 12 mg (higher dose) or 6 mg (standard dose) of intravenous dexamethasone once daily for up to 10 days. Four days of dexamethasone treatment before randomization was permitted, and the number of days on dexamethasone before randomization was included in the intervention period (ranging from 6 to 10 days).

The present sub-study included trial participants enrolled at trial sites in Denmark in the Capital Region and Region Zealand, including Rigshospitalet, Herlev and Gentofte Hospital, Amager and Hvidovre Hospital, North Zealand Hospital, Bispebjerg and Frederiksberg Hospital, Zealand University Hospital, Koege and Slagelse Hospital, who had at least one PG measurement after randomization.

#### Data collection

Data were obtained from the Remdesivir database, a comprehensive Danish database of prospectively collected health records from patients treated with Remdesivir during hospitalization for COVID-19 [17, 18]. Missing data were retrospectively collected. Data included demographic variables such as age and sex, chronic comorbidities including cardiovascular disease, hypertension, chronic obstructive pulmonary disease, and current malignancy, baseline oxygen requirements (defined as ambient air, low-flow oxygen, high-flow oxygen, or mechanical ventilation within 24 h of admission), any measured glycated hemoglobin A1c (HbA1c), PG measurements throughout hospitalization, diabetes status, glucose-lowering treatment before and during hospitalization including sodium-glucose transport protein 2 inhibitor, glucagon-like-peptide 1 receptor agonist, dipeptidyl peptidase 4 inhibitors, sulfonylureas, metformin, and units of rapid-acting insulin for the first 14 days, intensive care admission, mechanical ventilation, and all-cause mortality at day 90.

#### Definitions

Diabetes status was grouped into three categories by HbA1c levels according to the American Diabetes Association [19]: Normoglycemia (HbA1c < 39 mmol/mol), Prediabetes (39 mmol/mol ≤ HbA1c ≤ 47 mmol/mol), and Diabetes consisting of either (1) a known diagnosis of diabetes or glucose-lowering treatment at the time of admission; or (2) HbA1c ≥ 48 mmol/mol. The HbA1c closest to admission was considered.

Hyperglycemic events were defined as at least two PG measurements ≥ 11.1 mmol/L, which corresponds to the general threshold for diabetes. Severe hyperglycemic events were defined as one PG ≥ 20 mmol/L.

PG levels were collected several times per day for each patient. The average for each patient per day was considered for the analysis of glycemic levels.

Glycemic coefficient of variation (CV) in the two treatment groups was measured on each day within the first six days after study inclusion.

### Statistical analysis

Baseline characteristics of the study population are presented with descriptive statistics stratified by higher vs. standard doses of dexamethasone.

The time-to-event analyses for reaching a hyper- or hypoglycemic event, for reaching an accumulated daily dose of rapid-acting insulin of 0, 20, and 40 IU within the two treatment groups were computed using competing event analysis by the Fine-Gray subdistribution hazard model accounting for death and discharge as competing events [20]. Participants were followed from treatment allocation until a hyperglycemic or severe hyperglycemic event, death, discharge, or end of the intervention period after a maximum of 10 days of dexamethasone treatment. End of the intervention period of other causes than  discharge or death were censored. Gray’s test was performed to test the overall difference in the competing events between the groups. The model was adjusted for age, diabetes status, mechanical ventilation, and hospital site. An interaction term between the diabetes group and allocation was implemented.

Two linear mixed models were used to estimate average PG levels during two periods: pre-dexamethasone (three to one days before starting dexamethasone) and during dexamethasone (zero to five days after randomization) to account for the within-patient correlation over time [21]. PG levels were analyzed after randomization for a maximum of six days, as some participants started the treatment before allocation (up to four days). The dexamethasone period before randomization was not considered in the model. However, a measure was made to adjust for those that received dexamethasone before randomization. Days were treated as a categorical variable to capture PG changes over time. The model adjusted for age, diabetes status, mechanical ventilation, pre-randomization dexamethasone use, hospital site, and baseline PG, defined as the last measurement before dexamethasone started. For those on dexamethasone before allocation, the model assumed no cumulative treatment effect, as confirmed by prior analysis (Clausen et al., personal communication). An interaction term for allocation and diabetes status was included. Participants who changed wards were censored, and those discharged were artificially censored, with inverse probability weighting applied. Stabilized weights were derived from a pooled logistic regression adjusted for age, sex, comorbidities, and baseline oxygen needs. Standardized predicted PG values for the pre-and during-dexamethasone periods are provided. Confidence intervals (CI) were calculated using bootstrap resampling [22]. Supplementary Figure S1 provides a graphical overview of the study design.

The glycemic CV in the two treatment groups was calculated as $100 \times \frac{Standard\, deviation\, (G)}{mean\, glucose\, (G)}$, where G is the sum of all the glucose measurements within the period.

Statistical analyses were performed using R (version 4.3.2). The mixed model analysis was performed with the package *mmrm* [23], and the competing risk analysis was performed with the package *cmprsk* [24].

### Subgroup analysis

Subgroup analysis of participants in the higher and standard dose of dexamethasone group stratified by diabetes status was conducted.

## Results

Of the 344 participants randomized at the seven trial sites, six were excluded due to a lack of PG measurement, ten because they received more than four days of dexamethasone before allocation, two because they were transferred to terminal care, one due to *Pneumocystis jirovecii* pneumonia, and four because they had missing data on dexamethasone treatment, yielding a total of 321 participants. Of 30,816 PG measurements throughout hospitalization, 24,511 were done during dexamethasone treatment.

Clinical characteristics of participants based on treatment group are outlined in Table 1.

**Table 1:** Baseline characteristics in participants randomized to 12 mg or 6 mg of dexamethasone

	12 mg n = 170	6 mg n = 151
Age, median (IQR)	65 (58, 76)	67 (58, 74)
Females, n (%)	52 (31%)	41 (27%)
Comorbidities, n (%)		
Hypertension	76 (45%)	78 (52%)
CVD	50 (29%)	49 (32%)
COPD	13 (7.6%)	15 (10%)
Cancer	17 (10%)	7 (4.6%)
Diabetes group, n (%)		
Normoglycemia	33 (20%)	30 (21%)
Prediabetes	79 (48%)*	49 (34%)*
Diabetes	53 (32%)*	66 (46%)*
*Missing*	*5*	*6*
Glucose-lowering treatment at admission		
> 1 glucose lowering treatment	30 (18%)	39 (26%)
Insulin	4 (2.4%)*	14 (9.3%)*
Metformin	25 (15%)	32 (21%)
GLP1-receptor agonist	6 (3.5%)	7 (4.6%)
SGLT2-inhibitor	7 (4.1%)	12 (7.9%)
DPP4-inhibitor	8 (4.7%)	11 (7.3%)
Sulfonylureas	0 (0%)	2 (1.3%)
Baseline oxygen requirements**, n (%)		
Ambient air	21 (12%)	19 (13%)
Low flow	54 (32%)	54 (36%)
High flow	76 (45%)	58 (38%)
Mechanical ventilation	19 (11%)	20 (13%)

Normoglycemia: HbA1c < 39 mmol/mol, Prediabetes: 39 mmol/mol ≤ HbA1c ≤ 48 mmol/mol, Diabetes: HbA1c ≥ 48 mmol/mol, a known diagnosis of diabetes or un glucose-lowering treatment at time of admission IQR: Interquartile range

^*^p ≤ 0.05

^**^Most severe within 24 h of admission

There were 170 participants in the higher dose group and 151 participants in the standard dose group. In general, the clinical characteristics of the two groups were similar, with a median age of 66 years (95% CI [58–75]) and most being male (71%). All comorbidities were similar between the groups, except for diabetes, which was less common in the higher dose group compared to the standard dose group (p = 0.026), while prediabetes was more prevalent in the higher dose group (p = 0.029). Accordingly, insulin treatment before hospitalization (p = 0.014) was more prevalent in the standard dose group. There were similar proportions of normoglycemia in the two groups. Table 2 presents the clinical characteristics during hospitalization, including glucose-lowering treatment and clinical outcomes in participants randomized to higher vs. standard doses of dexamethasone.

**Table 2:** Clinical characteristics during hospitalization, including glucose-lowering treatment, dexamethasone treatment prior to randomization, and clinical outcome in participants randomized to 12 mg or 6 mg dexamethasone treatment

	12 mg N = 170	6 mg N = 151
Accumulated IE rapid-acting insulin during hospitalization, median (IQR)	48 (0, 159)	72 (0, 171)
Accumulated IE long-acting insulin during hospitalization, median (IQR)	0 (0, 0)	0 (0, 0)
Glucose-lowering drug prescribed at discharge, n (%)	11 (6.5%)	7 (4.6%)
Treated with dexamethasone prior to randomization, n (%)	86 (51%)	87 (58%)
Days of dexamethasone prior to randomization, median (IQR)	2 (1–3)	1 (1–2)
Intensive care unit admission, n (%)	129 (76%)	106 (71%)
*Missing*	*1*	*1*
Mechanical ventilation, n (%)	91 (54%)	73 (48%)
*Missing*	*2*	*0*

DPP4: dipeptidyl peptidase-4, GLP-1: glucagon-like peptide 1, IQR: Interquartile range, SGLT2-inhibitor: sodium-glucose transport protein 2

Treatment with dexamethasone before randomization was similar in the two groups (p = 0.251). There were no differences in oxygen requirements within the first 24 h of admission. While 12% required mechanical ventilation, another 12% did not require any oxygen at admission. However, all patients had progressed to at least 10 L of supplemental oxygen as per study inclusion criteria. Most were treated in the ICU, and nearly half required mechanical ventilation during hospitalization. Ninety-eight (30%) participants died within 90 days.

There were no differences in glycemic variability between the two dexamethasone groups, as assessed by coefficient of variation on the first six days after study inclusion (Table 3).

**Table 3:** The glycemic coefficient of variation during the first 6 days after study inclusion in participants randomized to 12 mg or 6 mg of dexamethasone treatment

		12 mg N = 170	6 mg N = 151
Day 1	CV, %, IQR	18 (11–25)	18 (13–25)
	*Missing*	*4*	*5*
Day 2	CV, %, IQR	18 (12–25)	19 (14–28)
	*Missing*	*7*	*3*
Day 3	CV, %, IQR	19 (14–28)	20 (13–27)
	*Missing*	*7*	*4*
Day 4	CV, %, IQR	21 (15–27)	21 (15–29)
	*Missing*	*10*	*4*
Day 5	CV, %, IQR	23 (16–31)	26 (20–33)
	*Missing*	*8*	*3*
Day 6	CV, %, IQR	22 (15–29)	23 (17–31)
	*Missing*	*11*	*8*

CV: Coefficient of variation; calculated as $100 \times \frac{Standard deviation (G)}{mean glucose (G)}$, where G is the sum of all the glucose measurements within the trial period

IQR: Interquartile range

Hyperglycemic events

On the sixth day, the cumulative incidence of a hyperglycemic event, defined as two PG measurements > 11.1 mmol/L, was 89% (95% CI 83–93%) in the higher dose group and 87% (81–92%) in the lower dose group. The incidence of a severe hyperglycemic event, defined as one PG measurement > 20.0 mmol/L, was 43% (35–51%) in the higher dose group and 34% (27–42%) in the standard dose group. The cumulative incidence of a hypoglycemic event (PG < 3.8 mmol/L) was 3% (0.7%−5%) in the higher dose group and 5% (2–8%) in the standard dose group. Regarding insulin use, the cumulative incidence of daily insulin use exceeding 20 IE was 46% (38–53%) in the higher dose group and 54% (45–61%) in the standard dose group. For daily insulin use exceeding 40 IE, the cumulative incidence was 25% (18–32%) in the higher dose group and 30% (23–38%) in the standard dose group.

**Fig. 1:**
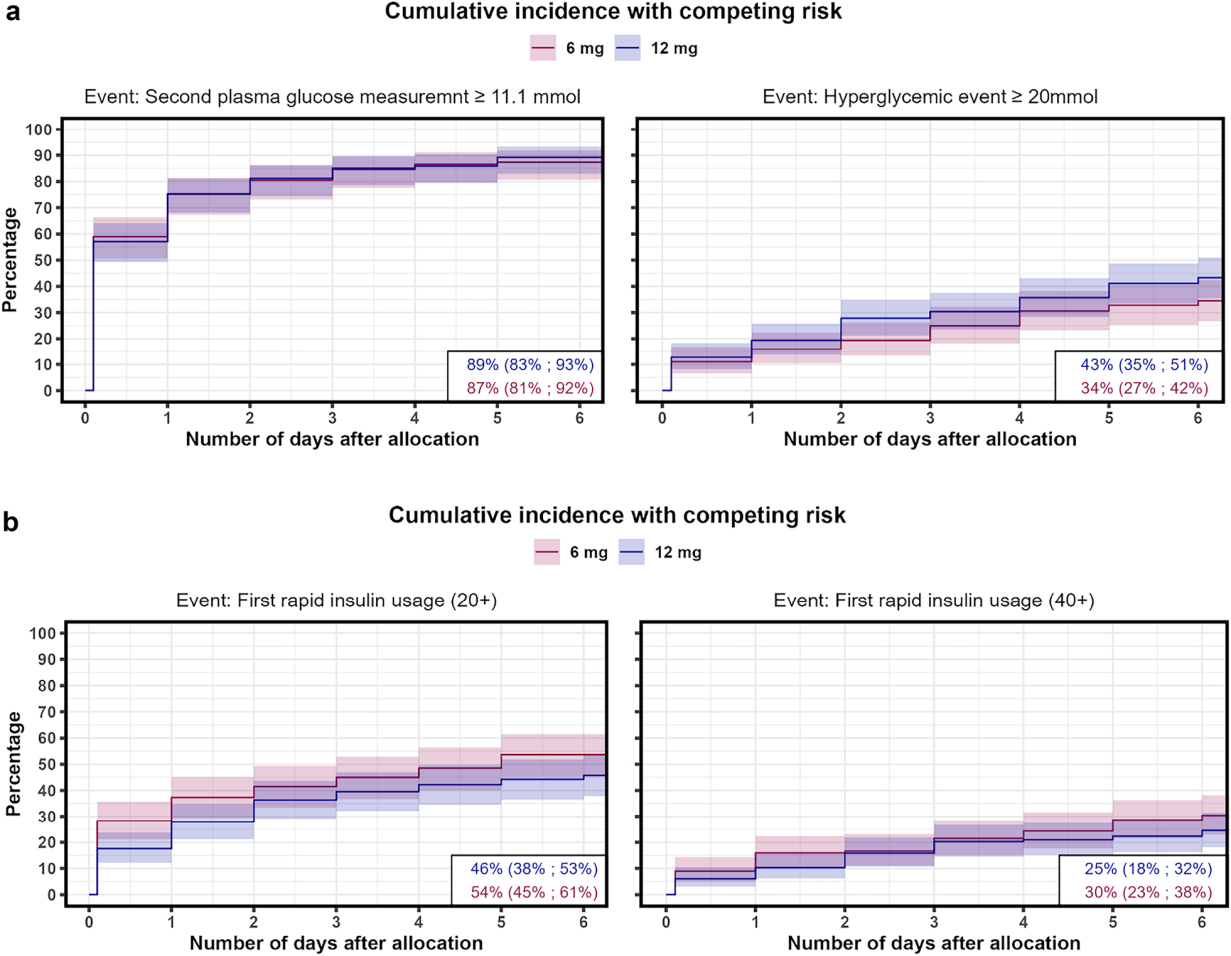
**a** Cumulative incidence functions for a hyperglycemic event (two PG measurements ≥ 11.1 mmol/L) or a severe hyperglycemic event (≥ 20 mmol/L) after allocation to 12 mg vs. 6 mg of dexamethasone in patients with COVID-19 and severe hypoxemia. **b** Cumulative incidence functions for daily use of ≥ 20 IE or ≥ 40 IE rapid-acting insulin after allocation to 12 mg vs. 6 mg of dexamethasone in patients with COVID-19 and severe hypoxemia

**Fig. 2:**
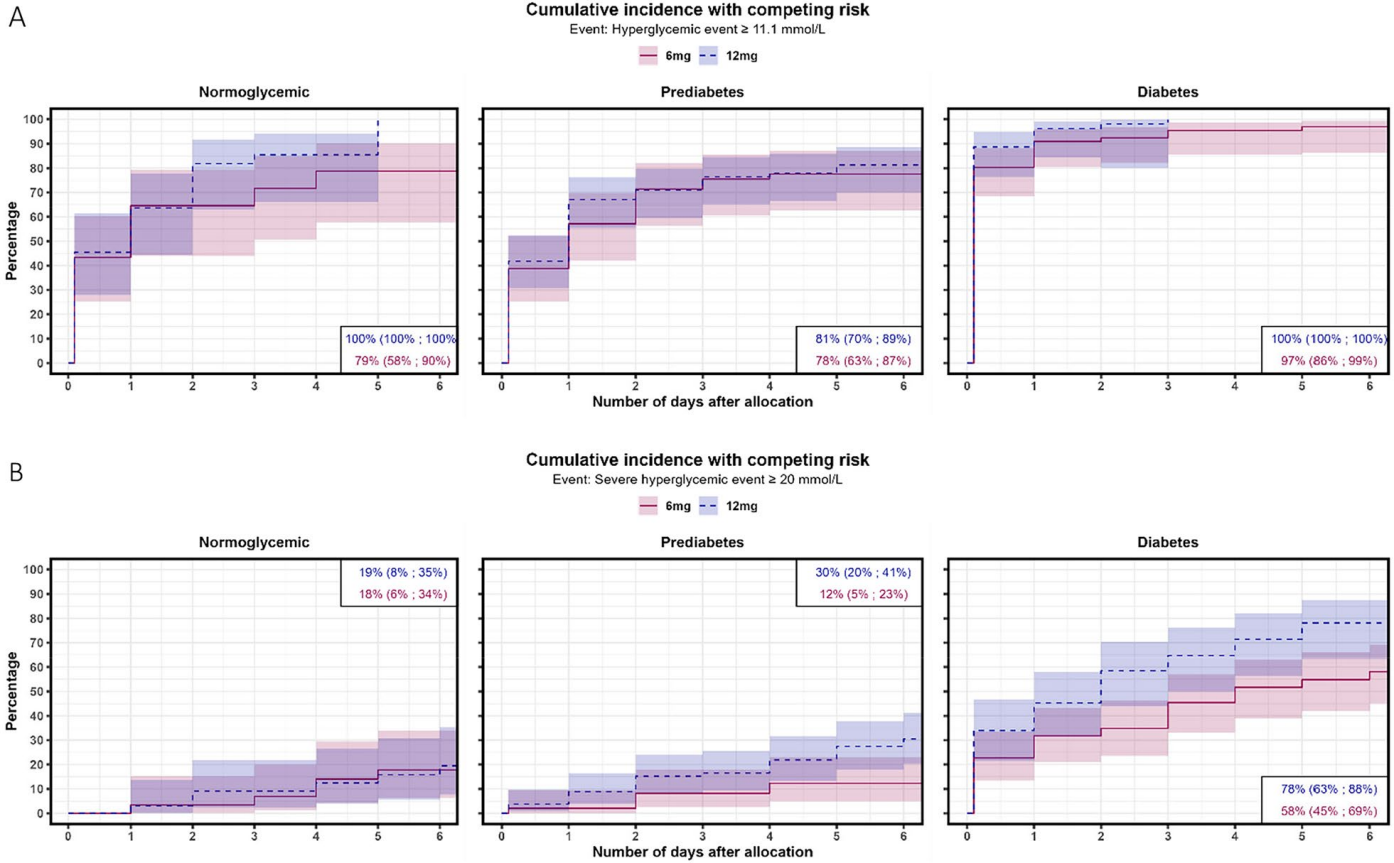
Cumulative incidence functions for a hyperglycemic event (**A**) (two PG measurements ≥ 11.1 mmol/L) or a severe hyperglycemic event (**B**) (≥ 20 mmol/L) within participants allocated to 12 mg or 6 mg of dexamethasone treatment stratified by diabetes status. The diabetes groups were defined as: Normoglycemia: HbA1c < 39 mmol/mol, prediabetes: HbA1c 39–47 mmol/mol, diabetes: HbA1c ≥ 48 mmol/mol, a known diagnosis of diabetes or on glucose-lowering treatment prior to admission

The cumulative incidences of time-to a hyperglycemic or a severe hyperglycemic event or of time-to-insulin use in the higher and standard dose groups are shown in Figs. 1a, b and 2a, b for the subgroup analysis.

There was no difference in events between groups in the unadjusted analysis. Upon adjustment for age, diabetes status, hospital site, and mechanical ventilation, allocation to higher dose dexamethasone was associated with a severe hyperglycemic event of 20 mmol/L (sHR 1.76 (95% CI [1.22–2.54], p = 0.003)), but not a hyperglycemic event of 11.1 mmol/L (sHR 1.13 (95% CI [0.67–1.88], p = 0.642)). There was no overall interaction between allocation and diabetes group (p = 0.849).

There was no difference in hypoglycemic events (≤ 3.8 mmol/L) between the higher and standard dose groups in crude or adjusted analysis. Additionally, there was no difference in insulin use in the time-to-event analysis of daily use of ≥ 0 IE, ≥ 20 IE, or ≥ 40 IE between the two groups (Supplementary Table E1).

**Fig. 3:**
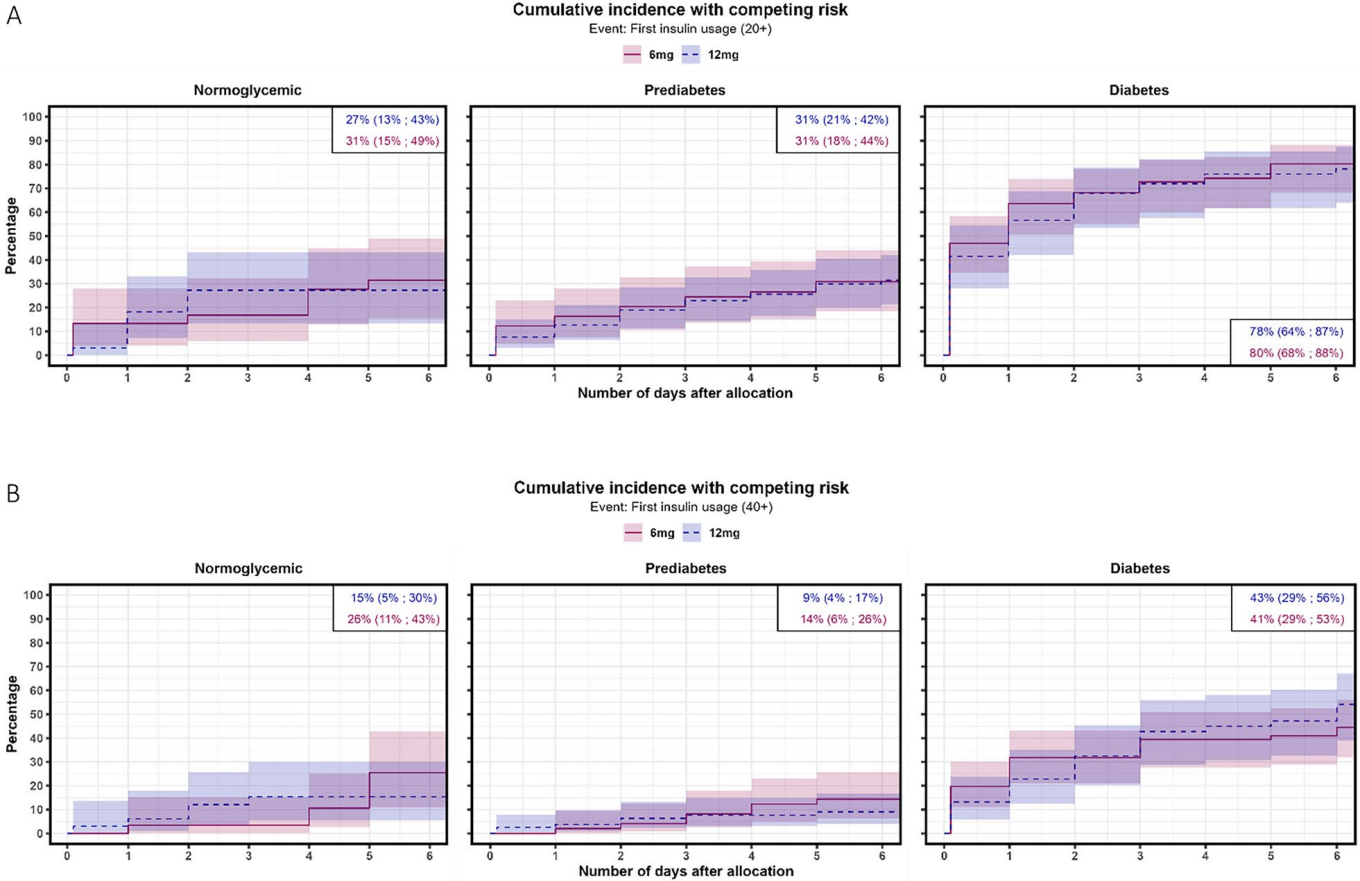
Cumulative incidence functions for daily insulin use of ≥ 20 IE (**A**) or ≥ 40 IE (**B**) within dexamethasone treatment in participants treated with 12 mg or 6 mg of dexamethasone stratified by diabetes status. The diabetes groups were defined as: Normoglycemia: HbA1c < 39 mmol/mol, prediabetes: HbA1c 39–47 mmol/mol, diabetes: HbA1c ≥ 48 mmol/mol, a known diagnosis of diabetes or on glucose-lowering treatment prior to admission. For visibility, confidence intervals are not shown

In the subgroup analysis (Figs. 2 and 3), diabetes and prediabetes were associated with a hyperglycemic event for higher versus standard doses of dexamethasone with an adjusted sHR of 1.73 (95% CI [1.10–2.72]), p = 0.018) and an sHR of 1.90 (95% CI [0.89–4.03], p = 0.097), respectively. Hazards of hyperglycemia did not differ for patients with normoglycemia (adjusted sHR of 1.43 (95% CI [0.46–4.46], p = 0.538)).

Plasma glucose levels before and after allocation to higher or standard dose dexamethasone.

**Fig. 4:**
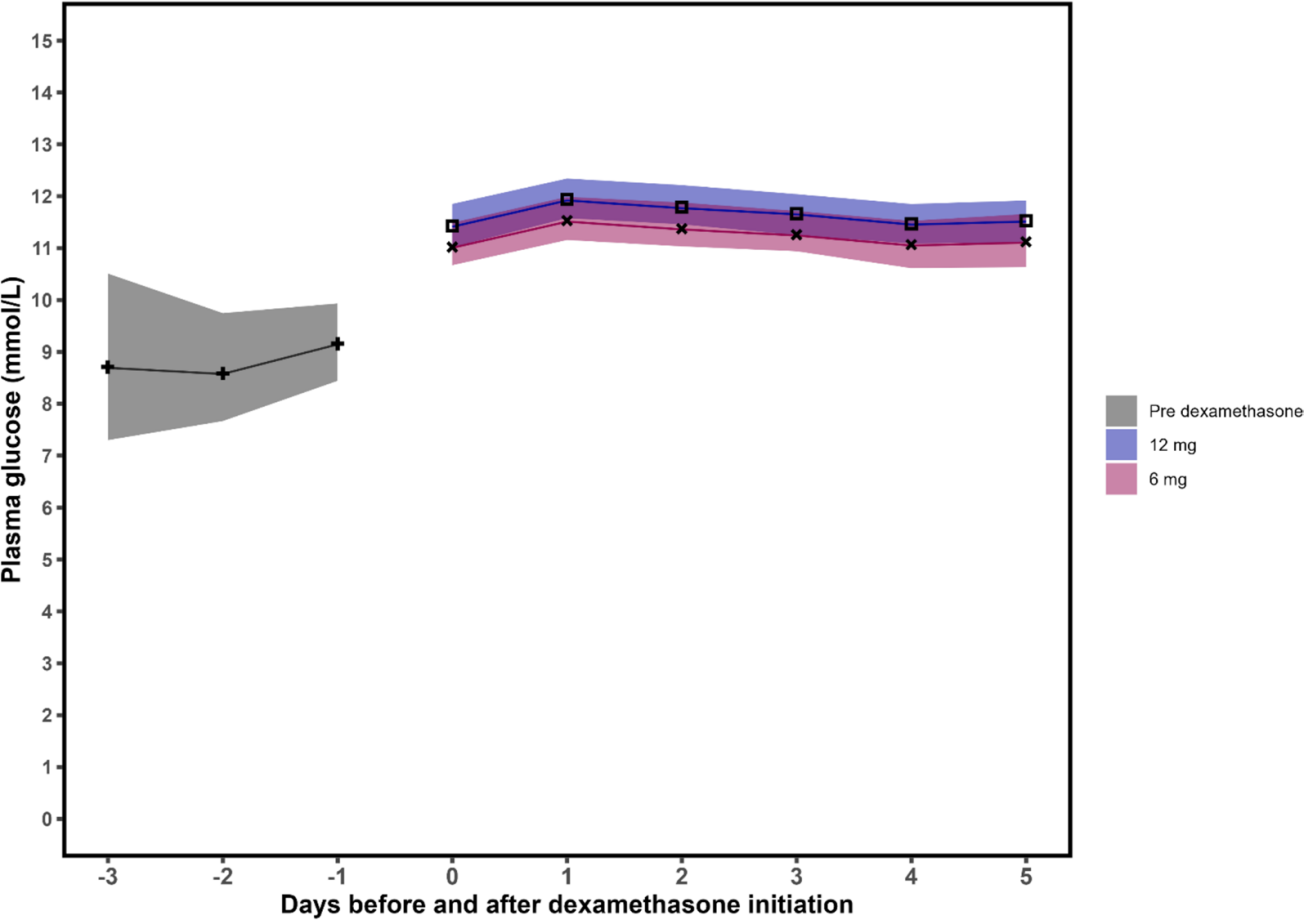
Glycemic variability from the pre-dexamethasone period to randomization to 12 mg or 6 mg of dexamethasone. The figure shows the predicted average plasma glucose values three days before and 6 days from randomization (day 0). The predictions were obtained by two linear mixed models adjusted for age, diabetes status, mechanical ventilation, baseline average plasma glucose, dexamethasone treatment prior to allocation, and hospital site with inverse probability of censoring weight for discharge. Confidence intervals were calculated by bootstrap resampling method

Figure 4 depicts differences in PG between groups.

Before dexamethasone start, the average PG was 9.1 mmol/L (95% CI [8.5–9.9]). After one day of either higher or standard dose dexamethasone, the average PG increased to 12.0 mmol/L (95% CI [11.6–12.3]) and 11.5 mmol/L (95% CI [11.2–12.0]), respectively, for an average PG increase of 0.5 mmol/L (95% CI [−0.2–1.4], p = 0.149) between groups. Participants with diabetes had a 3.0 mmol/L (95% CI [2.2–3.9], p < 0.001) higher PG compared to those with normoglycemia. The PG of those with prediabetes was not different compared to those with normoglycemia (0.3 mmol, 95% CI [− 0.3 to 1.5], p = 0.166).

Compared to the day of randomization (day 0), the 1st and 2nd day resulted in an increase in PG (0.5 mmol/L, 95% CI (0.3–0.8), p < 0.001 and 0.4 mmol/L [0.1–0.7], p < 0.05, respectively) in the higher dose group compared to the standard dose group. However, there was no effect on the 3rd, 4th, or 5th day after allocation.

## Discussion

In patients with COVID-19 and severe hypoxemia, we found that allocation to higher vs. standard dose dexamethasone was associated with a higher incidence of severe hyperglycemic events despite use of insulin. This increase was most pronounced in participants with diabetes. However, the average PG levels did not differ significantly between allocation groups.

Hyperglycemia is associated with more severe disease, including the development of ARDS and mortality in patients with COVID-19 [11–14]. One in three patients had hyperglycemia of 20 mmol/L in the two groups, with higher hazards in the higher dose group. However, the average PG levels (with respect to age, diabetes status, pre-allocation PG, pre-allocation dexamethasone use, and hospital site) and the coefficient of variation overlapped in the two groups, suggesting that despite the higher risk of severe hyperglycemia in the higher dose group, glucose-lowering treatments were effective in maintaining comparable average PG levels. There was no difference in hypoglycemic events between groups, and the incidence was also relatively low (3%−5%), indicating that the glucose-lowering regimen was not overly strict.

Over the years, randomized controlled trials (RCTs) have sought to establish the optimal PG threshold for critically ill patients, yielding varying results [25, 26]. Van den Berghe initially reported reduced mortality with a PG target range of 4.4 to 6.1 mmol/L [27]. The subsequent NICE-SUGAR trial found that a higher threshold of 10.0 mmol/L was associated with lower mortality and a reduction in severe hypoglycemia compared to tighter control between 4.5 and 6.0 mmol/L [26]. More recently, a large randomized clinical trial demonstrated similar outcomes between tight blood glucose control (4.1 to 6.6 mmol/L) and more liberal control (10.0 to 11.9 mmol/L) [28]. However, these trials included a broad spectrum of patients, particularly with a high prevalence of surgical cases, which limits comparability to populations of patients with medical illnesses treated with dexamethasone.

No specific PG threshold has been established for patients with COVID-19. An observational study of COVID-19 patients with diabetes reported that maintaining glycemic variability within 3.9 to 10.0 mmol/L was associated with significantly lower mortality compared to those with poorly controlled PG (> 10.0 mmol/L) [29]. Although this study is one of the larger observational studies, it is limited by confounding by indication. In our study, both groups had an average PG level above 10 mmol/L, and nearly all patients had at least two PG measurements exceeding 11 mmol/L during the study. Further randomized controlled trials are needed to determine the optimal glucose-lowering regimen and PG thresholds in severely ill patient, including those with respiratory tract illnesses.

There was an imbalance in our substudy of the COVID STEROID trial in the proportion of individuals with diabetes (32% in the higher dose group and 46% in the standard dose group). The observed imbalance likely contributed to a lower incidence of hyperglycemia, in the higher dose group, which may explain the absence of a significant difference in hyperglycemic events prior to adjustment for diabetes status.

Several RCTs have compared higher vs. standard dose dexamethasone treatment in COVID-19. Of these, five studies reported hyperglycemia as adverse events [30–34]. Three studies found no differences in hyperglycemia between higher dose (17–20 mg) and standard dose (6 mg) groups [30, 31, 33], while two observed a higher incidence of hyperglycemia in those receiving higher dose dexamethasone (20–24 mg) [32, 34]. The three studies that found no differences defined hyperglycemia as requiring insulin [30, 31, 33], which aligns with the findings of our study, where we did not observe any differences in insulin treatment between the groups. The RECOVERY trial, which did find group differences in hyperglycemia, also defined it as requiring insulin [32]. As this trial included the largest number of patients, we expect that the lack of positive findings regarding differences in insulin-treatment in the present study could be ascribed to issues with statistical power. Lastly, the trial using the highest dose of dexamethasone (24 mg/day), reported a higher rate of hyperglycemia (≥ 10 mmol/L) [34]. Neither of the trials accounted for the possible effect of prediabetes or diabetes on the risk of hyperglycemia thereby possibly underestimating the hyperglycemic effects.

Studies on healthy individuals have shown that there is a clear dose–response relationship between glucocorticoids and plasma glucose [35]. However, this relationship was not as evident in our study. We hypothesize that this may be due to the severity of illness in our population. The stress of severe disease may have already led to impaired insulin sensitivity and increased endogenous glucose production, thereby reducing the additional effects of dexamethasone on glucose metabolism.

To our knowledge, this is the first study to detailed analyze the glucose variability following high- and standard dose dexamethasone treatment. We have previously reported that patients with diabetes were at greater risk of experiencing hyperglycemia (Clausen et al., personal communication). The present findings further adds that higher doses of dexamethasone pose even greater risks in terms of severe hyperglycemia.

Given the elevated PG levels and increased risk of hyperglycemia associated with higher doses of dexamethasone, strategies to reduce glucose variability—such as corticosteroid-sparing alternatives like baricitinib or tocilizumab for COVID-19—could be explored in future trials. However, due to the high cost and limited availability of these alternatives, it may be more practical to focus on optimizing insulin regimens and determining the ideal PG threshold in patients with respiratory illness receiving corticosteroids. This is particularly important given the potential benefits of higher doses of dexamethasone, as suggested by the Bayesian analysis [4]. These approaches should be evaluated in randomized trials that are sufficiently powered to detect differences in mortality.

The strengths of this study include the comprehensive data collection, including a high number of PG measurements taken within the study period, allowing for a detailed examination of the PG levels after randomization. Most participants in this study had HbA1c determined, allowing for stratification and adjustment according to diabetes status.

This study does have limitations. Firstly, a small proportion of participants were excluded in the time-to-event analysis and the linear mixed model due to missing values. Although they were few, it creates a risk of selection bias, as these patients may have been more well compared to those included in the analysis. Secondly, any HbA1c measurement was permitted. Seventy percent of HbA1c measurements were taken within 60 days prior to admission or 7 days after admission, 12% were taken before this (with a median of −197 days before admission), and 12% were taken after (with a median of 32 days after admission). The latter two groups may have led to misclassification of diabetes status. Thirdly, the sample size was only about 1/3 of the total population limiting statistical power. Fourthly, the analysis was a post-hoc analysis, not predefined and had many outcome measures leading to the risk of type-1 errors.

## Conclusion

Higher dose dexamethasone was associated with a greater hazard of a severe hyperglycemic event, particularly in patients with diabetes, but with an average increase in PG like that of the standard dose group and similar needs of insulin. This suggests that while higher dose dexamethasone led to greater glucose variability, the overall dose-dependent relationship between corticosteroids and plasma glucose is less pronounced in severely ill patients.

## Supplementary Information

Additional file 1

## Data Availability

The datasets used and/or analysed during the current study are available from the corresponding author on reasonable request.

## Abbreviations

CI: Confidence Interval
COVID-19: Coronavirus disease 2019
HbA1c: Glycated hemoglobin A1c
PG: Plasma glucose
RCT: Randomized controlled trial
sHR: Subdistributional Hazard Ratio
