# Supplementary material for: Hyperglycemia and insulin use in patients with COVID-19 and severe hypoxemia allocated to 12 mg vs. 6 mg of dexamethasone: a secondary analysis of the COVID STEROID 2 randomized trial
Source: Ann Intensive Care. 2025 Jul 15;15:98. doi: 10.1186/s13613-025-01512-5 (PMC12263535; doi:10.1186/s13613-025-01512-5)

**Supplementary Table E1:** Time-to-event analysis in the 12 mg or 6 mg group. The model is adjusted on diabetes status, age, mechanical ventilation, and hospital site. Model estimates are subdistributional hazard ratios (sHR) with 95% confidence intervals (CI).

|  | Crude, sHR (95% CI) | p-value | Adjusted, sHR (95% CI) | p-value |
| --- | --- | --- | --- | --- |
| Hyperglycemic event |  |  |  |  |
| 6 mg | Ref | - | Ref | - |
| 12 mg | 1.02 (0.81-1.30) | 0.843 | 1.13 (0.67-1.88) | 0.642 |
| Severe hyperglycemic event (> 20 mmol/L) |  |  |  |  |
| 6 mg | Ref | - | Ref | - |
| 12 mg | 1.23 (0.87-1.73) | 0.244 | 1.76 (1.22-2.54) | 0.003 |
| Any daily insulin use (>0 IU)* |  |  |  |  |
| 6 mg | Ref | - | Ref | - |
| 12 mg | 0.97 (0.74-1.28) | 0.855 | 1.05 (0.79-1.39) | 0.727 |
| Accumulated daily rapid-acting insulin use > 20 IU |  |  |  |  |
| 6 mg | Ref | - | Ref | - |
| 12 mg | 0.80 (0.59-1.09) | 0.160 | 0.94 (0.63-1.40) | 0.751 |
| Accumulated daily rapid-acting insulin use > 40 IU |  |  |  |  |
| 6 mg | Ref | - | Ref | - |
| 12 mg | 0.74 (0.49-1.11) | 0.148 | 1.09 (0.65-1.83) | 0.758 |
| Hypoglycemic event |  |  |  |  |
| 6 mg | Ref | - | Ref | - |
| 12 mg | 0.60 (1.26-1.38) | 0.229 | 0.60 (0.24-1.54) | 0.601 |

IU: International units

* Those treated with insulin before admission were excluded from the analysis (N=18)

**Supplementary Figure S1:** Representation of hypothetical patients within the study.

Linear mixed effects model was employed with imputations by inverse probability of censoring weighting or within the model by the missing at random assumption. The shift from pre-dexamethasone (blue) to allocation-period (higher dose or standard dose) was analyzed. The standard-of-care dexamethasone period (orange) was adjusted for in the model.


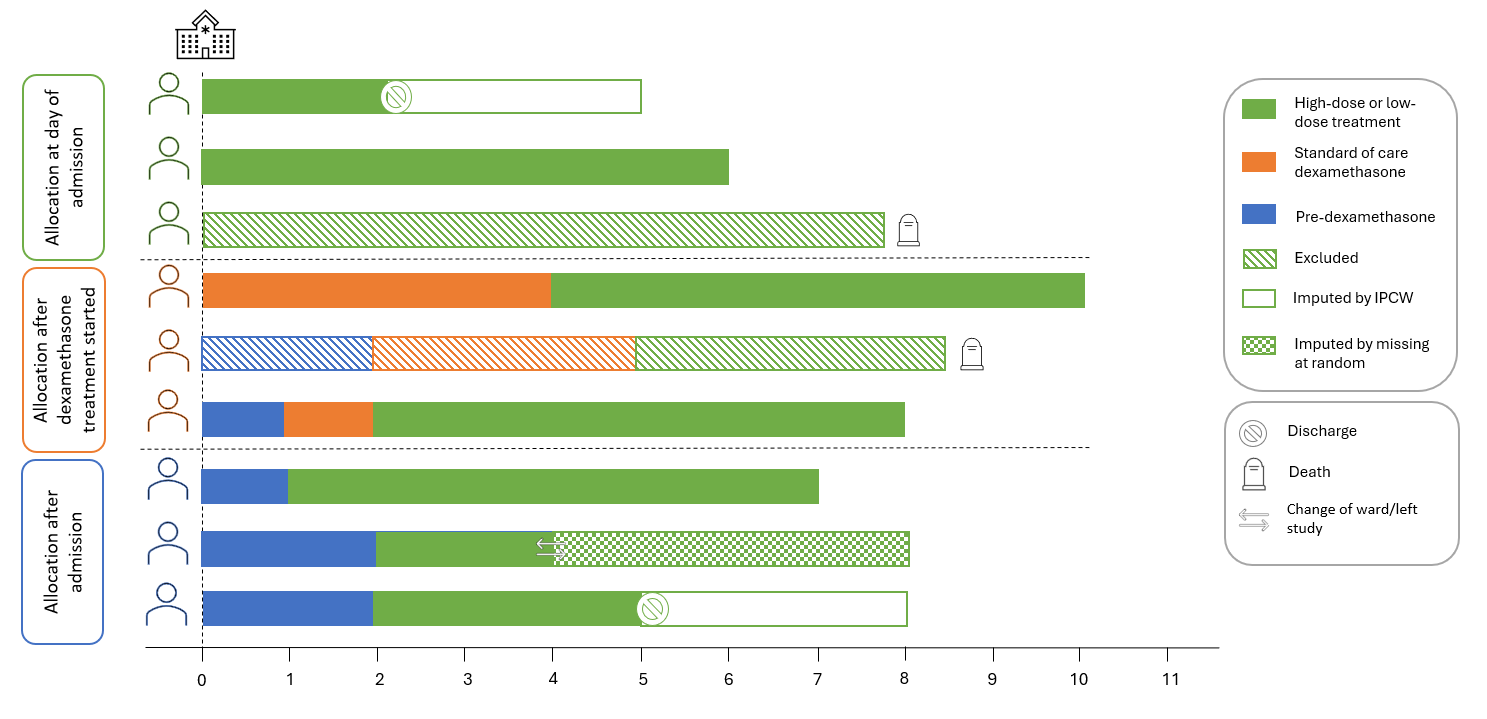

Supplement: Supplementary file 1 — Additional file 1 [file 13613_2025_1512_MOESM1_ESM.docx]
